# CHI3L1 Is Associated With TP53 Signaling and Promotes Papillary Thyroid Carcinoma Progression

**DOI:** 10.1002/cnr2.70553

**Published:** 2026-04-19

**Authors:** Fen Cai, Ya'nan Zhou, Yeran Yang, Meng Zhang, Xuan Zhang, Yan Chang, Shengcai Wang, Xinyuan Wu, Yongli Guo, Yongbo Yu, Xin Ni, Jiangqiao Geng

**Affiliations:** ^1^ Department of Otolaryngology, Head and Neck Surgery Hebei Children's Hospital, Hebei Medical University Shijiazhuang Hebei China; ^2^ Beijing Key Laboratory for Pediatric Diseases of Otolaryngology, Head and Neck Surgery, MOE Key Laboratory of Major Diseases in Children, Beijing Pediatric Research Institute, Beijing Children's Hospital, Capital Medical University, National Center for Children's Health (NCCH) Beijing China; ^3^ Department of Pathology Beijing Children's Hospital, Capital Medical University, National Center for Children's Health (NCCH) Beijing China; ^4^ Department of Otolaryngology, Head and Neck Surgery Beijing Children's Hospital, Capital Medical University, National Center for Children's Health (NCCH) Beijing China; ^5^ Biobank for Clinical Data and Samples in Pediatrics, Beijing Children's Hospital, Capital Medical University, National Center for Children's Health (NCCH) Beijing China

**Keywords:** CHI3L1, migration, papillary thyroid carcinoma, proliferation, TP53 pathway

## Abstract

**Background:**

Papillary thyroid carcinoma (PTC) is the most commonly diagnosed subtype of thyroid cancer and represents a highly prevalent form of endocrine malignancy.

**Aims:**

This study aimed to investigate the role and molecular mechanism of CHI3L1 in PTC progression.

**Methods and Results:**

CHI3L1 expression in PTC was analyzed using public datasets. Cell proliferation was assessed using the CCK‐8 assay and colony formation assay. Tumor growth was evaluated using nude mouse xenograft models. Cell invasion was evaluated using the transwell assay, while cell migration was assessed with the wound healing assay. Transcriptomic analysis was conducted to examine the molecular mechanism, and real‐time quantitative PCR was performed for gene expression validation. The findings revealed that CHI3L1 expression was upregulated in various cancers, mainly in PTC. Both in vitro and in vivo assays demonstrated that cell proliferation was suppressed when CHI3L1 was knocked down. Transcriptome sequencing indicated that CHI3L1 knockdown was associated with migration‐related pathways and the TP53 signaling pathway. Transwell assays showed reduced cell invasion upon CHI3L1 suppression, while wound healing assays demonstrated decreased cell migration. Following CHI3L1 silencing, real‐time quantitative PCR verified the overexpression of TP53‐related genes. Survival analysis further indicated a correlation between elevated CHI3L1 expression and reduced survival rates.

**Conclusion:**

This study identified that CHI3L1 was an oncogene in PTC and promotes tumor cell proliferation associated with downregulating the TP53 pathway. It provides new evidence supporting CHI3L1 as a potential molecular target for future therapeutic investigation in PTC.

## 1 Introduction

Papillary thyroid carcinoma (PTC) is the most commonly diagnosed subtype of thyroid cancer, which is an indolent tumor [1]. In recent years, PTC incidence has steadily increased [2]. According to the American Thyroid Association, most PTC patients have a favorable prognosis, with a mortality rate of 1%–6.5%, but the recurrence rate reaches 15%–30% [3]. Therefore, elucidating the molecular mechanisms underlying PTC progression remains important for identifying potential therapeutic targets.

Genetic mutations, deletions, and alterations in gene expression play a critical role in tumor development. Several genes, including CITED1, CHI3L1, ODZ1, N33, SFTPB, and SCEL, have been stated to be upregulated in PTC [4]. In our previous study, we identified that Chitinase 3‐like 1 (CHI3L1) was highly expressed in PTC [5]. CHI3L1 is a 40‐kDa glycoprotein produced by various human cell types, including synovial, inflammatory, fibroblast‐like, and cancer cells [6, 7, 8, 9, 10]. It plays a crucial role in cellular processes such as inflammation, proliferation, apoptosis, differentiation, and metastasis [7, 11, 12, 13, 14]. Notably, CHI3L1 expression increases during Th2‐mediated inflammatory responses triggered by stimuli [15]. CHI3L1 promotes cancer cell growth by activating multiple signaling pathways [16, 17, 18] and influences the differentiation of CD4+ T cells into Th1 cells [19]. Evidence suggests that CHI3L1 contributes to carcinogenesis by altering the extracellular matrix (ECM) to protect cancer cells from apoptosis [20]. In various cancers, CHI3L1 has been associated with metastasis, including lung cancer, melanoma, and breast cancer [21, 22]. However, despite its known roles in other cancers, the function of CHI3L1 in PTC is still not fully understood.

Cheng et al. previously conveyed that CHI3L1 is associated with the development and recurrence of thyroid cancer [23]. Furthermore, high expression levels of CHI3L1 were linked to a poor prognosis in patients with PTC [24]. However, the specific function and molecular mechanisms underlying CHI3L1 in PTC remain unclear. Therefore, this study aims to elucidate the relationship between CHI3L1 and PTC. Specifically, we examined the functional effects of CHI3L1 on cell proliferation, invasion, and migration. Using transcriptomics and PCR analysis, we identified the signaling pathways through which CHI3L1 promotes cell proliferation. Clinically, the prognostic role of CHI3L1 was explored in patients with PTC.

## 2 Materials and Methods

### 2.1 Data Processing and Gene Expression Analysis

To examine the difference in CHI3L1 expression between tumor and normal tissues, PTC datasets were downloaded from the TCGA database (https://portal.gdc.cancer.gov/) and GTEx database (https://commonfund.nih.gov/GTEx). To further validate CHI3L1 expression, the GSE33630, GSE27155, GSE3678, and GSE3467 datasets were obtained from the GEO website (www.ncbi.nlm.nih.gov/geo). Data analysis and creating visualizations were performed using R software (version 4.2.2).

### 2.2 Cell Lines and Cell Culture

The TPC‐1, KTC‐1, and BCPAP cell lines were obtained from the Cell Resource Center (IBMS, CAMS/PUMC, China) and authenticated by STR profiling. The cell lines were cultured in RPMI 1640 medium (Gibco, China) complemented with 10% fetal bovine serum (FBS, Gibco, USA) and penicillin–streptomycin (Gibco, USA) at 37°C under 5% CO2.

### 2.3 Lentivirus Infection

To create a stable transfection cell line, cells were seeded in a six‐well plate at a density of 1 × 10^5^ and transfected manually. The lentivirus for CHI3L1 knockdown was supplied by Genechem (Shanghai, China). The following sequences were used as targets: CAAGGAAATGAAGGCCGAATT (sh‐CHI3L1#1), CCTGACAGATTCAGCAACACT (sh‐CHI3L1#2) and TTCTCCGAACGTGTCACGT (sh‐NC) (Shanghai Genechem Co. Ltd.). After 48 h, the cells were cultured in RPMI containing 0.5 μg/mL puromycin (LABLEAD, China) for selection. The transfected cells were subsequently used for further experiments.

### 2.4 Cell Proliferation Assay

Cell proliferation was assessed using the CCK‐8 assay (Beyotime, China). Cells were seeded in 96‐well plates with a density of 3 × 10^3^ cells per well. The CCK‐8 reagent (20 μL/well) was added at 24, 48, 72, 96, and 120‐h time points. After incubating for 3 h, the absorbance was measured at 450 nm using a microplate reader (CLARIO star, Germany).

### 2.5 Colony Formation Assay

To evaluate the clonogenic potential of the cells upon CHI3L1 knockdown, a colony formation assay was conducted. Cells were seeded in six‐well plates at a density of 600 cells per well and cultured for 10 days to allow colony formation. The medium was replaced every 3–4 days. After the incubation period, the cell colonies were fixed with 4% paraformaldehyde for 15 min at room temperature. Subsequently, the colonies were stained with 0.5% crystal violet for 30 min. Excess stain was gently removed with distilled water, and the plates were air‐dried before imaging. The number of colonies formed in each well was quantified using ImageJ software.

### 2.6 Wound Healing Assay

A wound‐healing assay was performed to evaluate the effect of CHI3L1 inhibition on cell migration. Cells were seeded in six‐well plates for 24 h and a sterile 200 μL pipette tip was used to create a straight scratch in the cell monolayer. Detached cells and debris were washed away with phosphate‐buffered saline (PBS). Wound images were captured at 0 and 48 h post‐scratch using a microscope (IX73, Olympus, Tokyo, Japan). Three random locations along the wound were selected for quantitative measurements, and the distance between wound edges was calculated. The extent of wound healing was expressed as the percentage of the initial wound width covered by migrating cells, using ImageJ software.

### 2.7 RNA‐Sequencing and Data Processing

RNA‐sequencing analysis was performed on TPC‐1 cells. Total RNA was extracted from three biological replicates of TPC‐1 cells, and gene expression levels were quantified using the Illumina NovaSeq 6000 platform, with gene expression measured as fragments per kilobase per million (FPKM). Differentially expressed genes (DEGs) were identified by applying a |log2FC| ≥ 1 and an adjusted *p* value < 0.05. Gene Ontology (GO) and Kyoto Encyclopedia of Genes and Genomes (KEGG) enrichment analysis were conducted to identify the biological processes associated with the DEGs, while Gene Set Enrichment Analysis (GSEA) was performed to explore broader pathway involvement. To investigate the relationship between DEGs and the TP53 pathway, overlapping genes were identified using a Venn diagram. The hallmark TP53 pathway gene set was obtained from the GSEA website (www.broad.mit.edu/gsea/index.html), and the results were visualized using heat maps.

### 2.8 RNA Extraction and qRT‐PCR Assay

Total RNA was extracted using the Direct‐zol RNA Miniprep kit (Zymo Research), and cDNA was synthesized using a reverse transcription kit (Takara). Quantitative real‐time PCR (qRT‐PCR) was conducted using SYBR Green Master Mix (BIO‐RAD) and the ViiA7 Real‐Time PCR System. GAPDH was used as the internal reference gene. Relative gene expression levels were calculated using the 2^−ΔΔCt^ method. Primer sequences for all target genes are provided in Table 1.

**TABLE 1:** The primer sequences used for all target genes.

The primer sequences		
Genes	Forward	Reverse
AEN	5′‐CAAGTGTGTGGCTATCGACTG‐3′	5′‐CACTCCAGCGGGTACGGTA‐3′
BAX	5′‐CCCGAGAGGTCTTTTTCCGAG‐3′	5′‐CCAGCCCATGATGGTTCTGAT‐3′
CASP1	5′‐TTTCCGCAAGGTTCGATTTTCA‐3′	5′‐GGCATCTGCGCTCTACCATC‐3′
CCND3	5′‐TACCCGCCATCCATGATCG‐3′	5′‐AGGCAGTCCACTTCAGTGC‐3′
CCNG1	5′‐GAGTCTGCACACGATAATGGC‐3′	5′‐GTGCTTGGGCTGTACCTTCA‐3′
CDH13	5′‐AGCGATGGCGGCTTAGTTG‐3′	5′‐CCCCGACAATCACGAGTTCTG‐3′
CDK1	5′‐AAACTACAGGTCAAGTGGTAGCC‐3′	5′‐TCCTGCATAAGCACATCCTGA‐3′
CDKN1A	5′‐CGATGGAACTTCGACTTTGTCA‐3′	5′‐GCACAAGGGTACAAGACAGTG‐3′
CHI3L1	5′‐GTGAAGGCGTCTCAAACAGG‐3′	5′‐GAAGCGGTCAAGGGCATCT‐3′
FAM162A	5′‐GTCAGGTCGCTTCAAAAAGGA‐3′	5′‐AGATAGCTGATCTTCACTCGCAT‐3′
FAS	5′‐TCTGGTTCTTACGTCTGTTGC‐3′	5′‐CTGTGCAGTCCCTAGCTTTCC‐3′
HBEGF	5′‐ATCGTGGGGCTTCTCATGTTT‐3′	5′‐TTAGTCATGCCCAACTTCACTTT‐3′
JUN	5′‐TCCAAGTGCCGAAAAAGGAAG‐3′	5′‐CGAGTTCTGAGCTTTCAAGGT‐3′
MDM2	5′‐GAATCATCGGACTCAGGTACATC‐3′	5′‐TCTGTCTCACTAATTGCTCTCCT‐3′
NUPR1	5′‐CTCTCATCATGCCTATGCCTACT‐3′	5′‐CCTCCACCTCCTGTAACCAAG‐3′
POLH	5′‐CTGGCACAAGTTCGTGAGTC‐3′	5′‐GCAACAAGTCTGCCGAGATAG‐3′
RAP2B	5′‐GTACGACCCGACCATCGAAG‐3′	5′‐TCGTCTACCGAGGCTTTGTTT‐3′
TGFA	5′‐AGGTCCGAAAACACTGTGAGT‐3′	5′‐AGCAAGCGGTTCTTCCCTTC‐3′
TP53	5′‐GAGGTTGGCTCTGACTGTACC‐3′	5′‐TCCGTCCCAGTAGATTACCAC‐3′
GAPDH	5′‐GAGTCAACGGATTTGGTCGT‐3′	5′‐TTGATTTTGGAGGGATCTCG‐3′

### 2.9 Transwell Migration Assay

Cell migration was assessed using Transwell chambers with 8 μm pore size filters (BD Biosciences, NJ, USA). Cells were starved in a serum‐free medium for 12 h and then seeded into the upper chamber at a density of 5 × 10^4^ cells per well in 250 μL serum‐free RPMI medium. The lower chamber contained 500 μL RPMI medium supplemented with 10% fetal bovine serum as a chemoattractant. After 24 h, non‐migrated cells on the upper side of the membrane were removed, and the migrated cells on the lower side were fixed with 4% paraformaldehyde for 30 min and stained with 2% crystal violet for 30 min. The membranes were rinsed with distilled water, air‐dried, and imaged under a microscope (IX73, Olympus, Tokyo, Japan). The number of migrated cells in three randomly selected fields per sample was quantified using ImageJ software.

### 2.10 Animal Experiments

Female BALB/c mice (8 weeks old) were gained from Beijing Huafukang Biotechnology Company. Mice were randomly divided into two groups (*n* = 10 per group). Group 1 mice were injected subcutaneously with 5 × 10^6^ BCPAP cells, while Group 2 mice received the same number of CHI3L1‐knockdown cells. Following tumor creation, tumor volumes, and mouse weights were thoroughly measured at regular intervals. Tumor volumes were calculated using the formula: volume = length × width^2^ × 0.5. Measurements were taken on days 5, 10, 15, 20, 25, 30, 35, and 40 days post‐injection.

### 2.11 Survival Analysis

CHI3L1 expression from the TCGA database (https://portal.gdc.cancer.gov/) was used to assess its prognostic significance in PTC patients. Patients were divided into high and low CHI3L1 expression groups based on the median expression level. Kaplan–Meier survival analysis was performed to compare patient survival between the two groups, and statistical significance was assessed using the log‐rank test. Survival curves were generated using R software (version 4.2.2).

### 2.12 Statistical Method

All statistical analyses were performed using R software (version 4.2.2). Data were presented as the mean ± standard deviation (SD). Student's *t*‐test or paired *t*‐test was used for comparisons between groups as appropriate. Kaplan–Meier survival analysis with the log‐rank test was used to evaluate differences in survival between groups. A *p* value < 0.05 was considered statistically significant.

## 3 Results

### 3.1 Elevated CHI3L1 Expression in Thyroid Cancer and PTC Subtype Across Multiple Datasets

**FIGURE 1:**
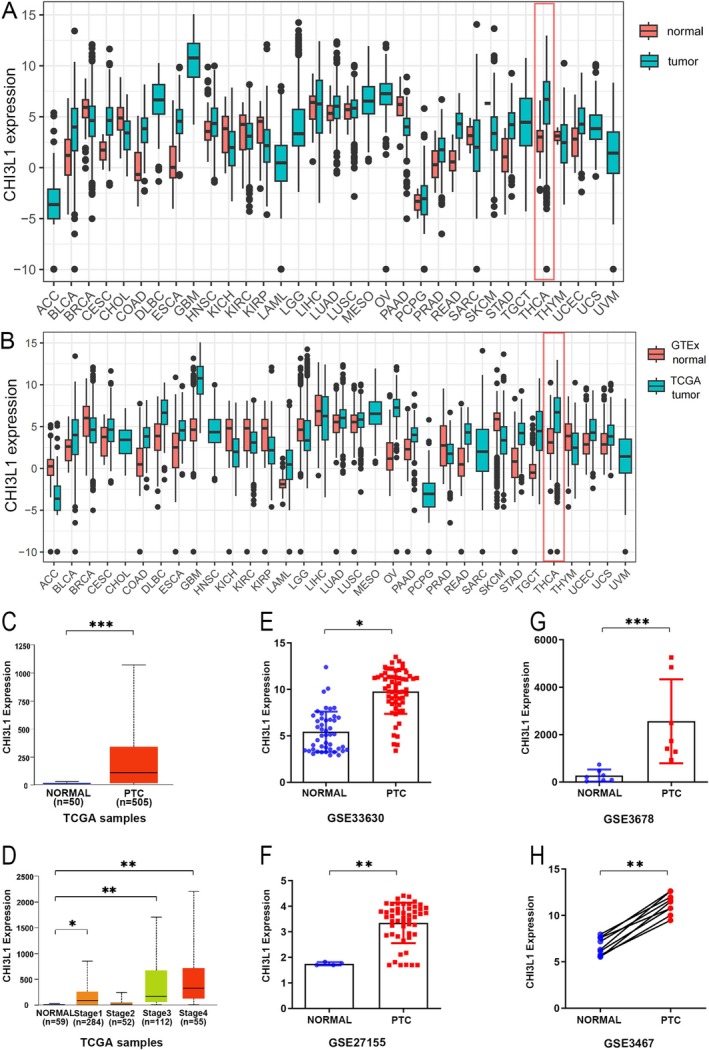
Comparative analysis of CH13L1 expression in various cancer and normal tissue samples. CHI3L1 expression levels contrasting normal tissue and papillary thyroid carcinoma (PTC) across different datasets. (A) CHI3L1 expression levels in tumor versus normal samples. (B) Comparison between GTEx normal samples and TCGA tumor samples, showing a significant increase in thyroid carcinoma. (C) Marked increase in CHI3L1 expression in PTC samples from TCGA. (D) TCGA samples stratified by disease stage. (E–H) External validation datasets (GSE36330, GSE27155, GSE3678, GSE3467) displaying consistent upregulation in PTC. Statistical significance denoted by asterisks (*p < 0.05, **p < 0.01, ***p < 0.001).

In this study, CHI3L1 expression was analyzed using the TCGA database, which contains data for 33 tumor types. To allow reliable tumor‐normal comparisons, 23 tumor types with available corresponding normal tissues were included in the present study. The results revealed that significantly elevated CHI3L1 was expressed in various tumor samples, particularly in thyroid cancer (Figure 1A). This finding was further validated by combining TCGA tumor samples with GTEx normal tissues, thereby confirming increased CHI3L1 expression in thyroid cancer (Figure 1B). Additionally, CHI3L1 mRNA expression in PTC was analyzed using designated datasets from TCGA on the UALCAN platform (https://ualcan.path.uab.edu/analysis.html). A comparison of 505 PTC samples with 59 normal samples showed a significantly higher CHI3L1 expression in the PTC group (Figure 1C). Moreover, CHI3L1 expression was markedly elevated in stages I, III, and IV of PTC compared to normal samples (Figure 1D). Further validation was performed using GEO datasets (GSE33630, GSE27155, GSE3678, GSE3467), which consistently demonstrated increased CHI3L1 expression in PTC (Figure 1E–H).

### 3.2 CHI3L1 Knockdown Inhibits Thyroid Cancer Cell Growth In Vitro and Tumor Development In Vivo

**FIGURE 2:**
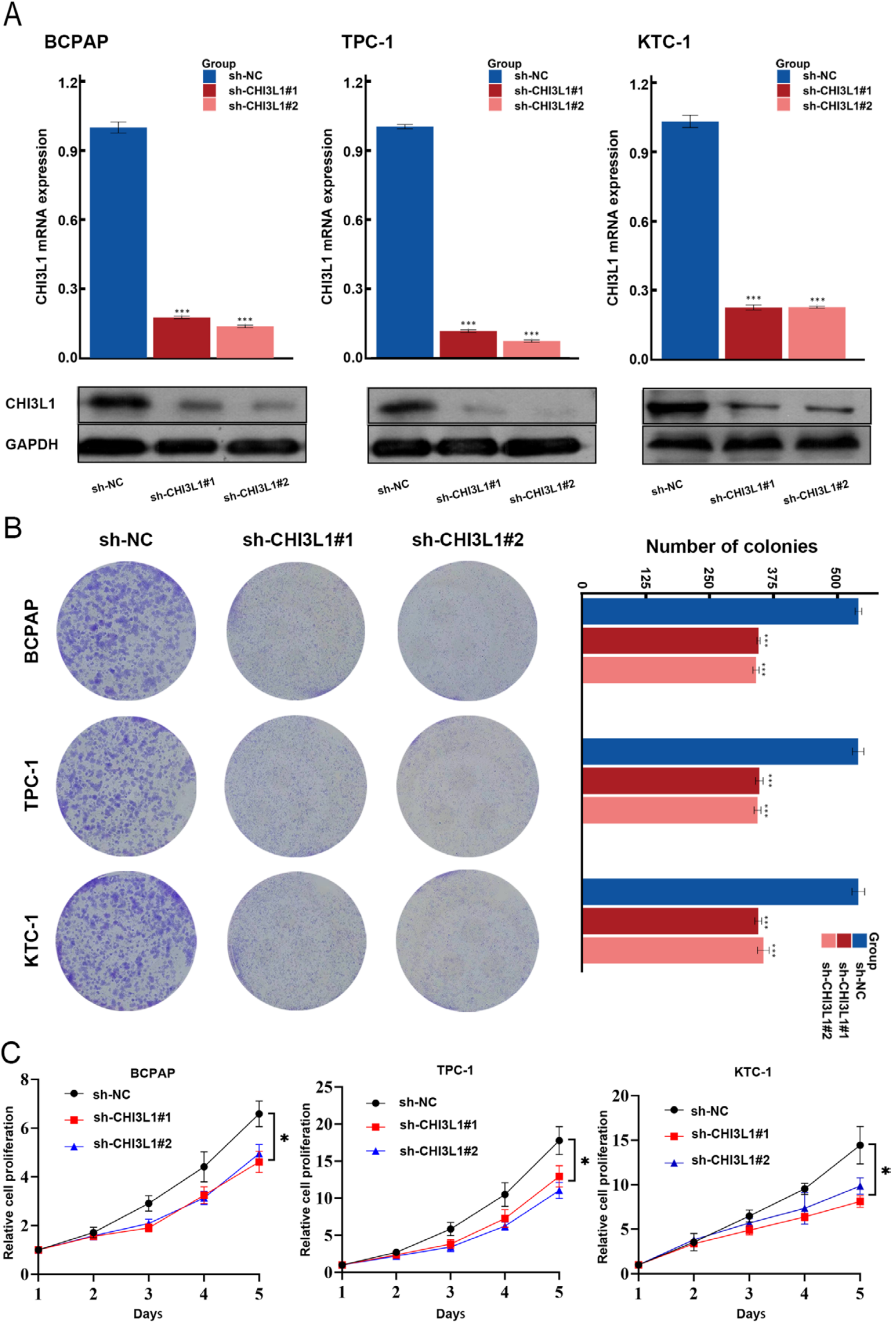
CHI3L1 knockdown inhibits thyroid cancer cell growth. (A) qPCR and western blot results confirming CHI3L1 knockdown efficiency in three thyroid cancer cell lines (BCPAP, TPC‐1and KTC‐1). (B) Colony formation assay results for BCPAP, TPC‐1, and KTC‐1 lines. (C) Cell proliferation assay results for BCPAP, TPC‐1, and KTC‐1 lines. (*p < 0.05, **p < 0.01, ***p < 0.001).

**FIGURE 3:**
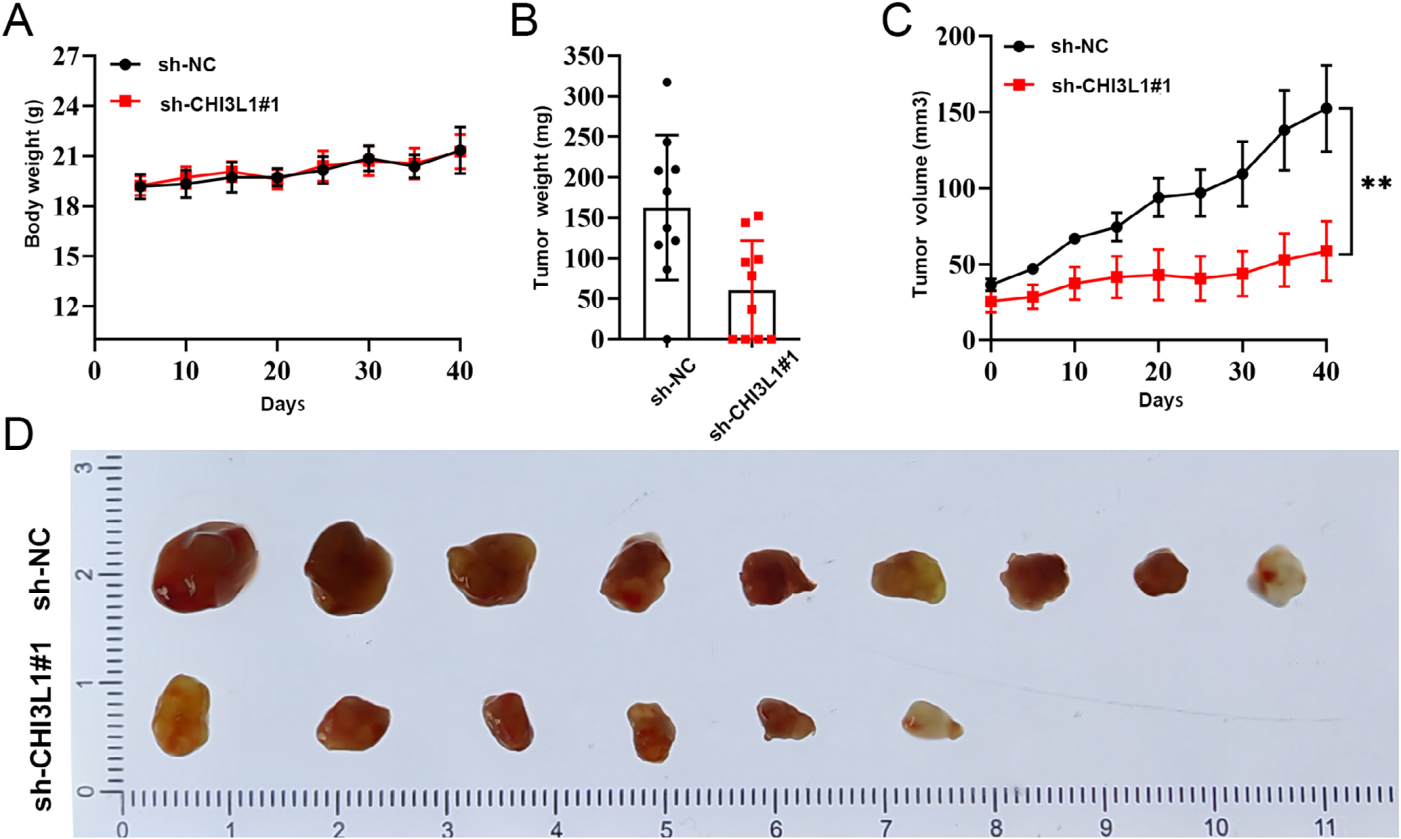
CHI3L1 knockdown inhibits tumor development in vivo. (A–D) Changes in body weights, tumor weights, and tumor volumes with CHI3L1 knockdown. Statistical significance is denoted by asterisks (*p < 0.05, **p < 0.01, ***p < 0.001).

Real‐time quantitative PCR and western blotting confirmed effective CHI3L1 knockdown in three thyroid cancer cell lines (Figure 2A). Colony formation and cell proliferation assays revealed significantly reduced growth among CHI3L1 knockdown cells (Figure 2B,C). Furthermore, in vivo xenograft experiments using BCPAP cell lines showed that both tumor volume and weight were significantly lower in the CHI3L1 knockdown group compared to the control group (Figure 3A–D).

### 3.3 Transcriptome Analysis Reveals CHI3L1 Regulates Cell Migration and Invasion in Thyroid Cancer

**FIGURE 4:**
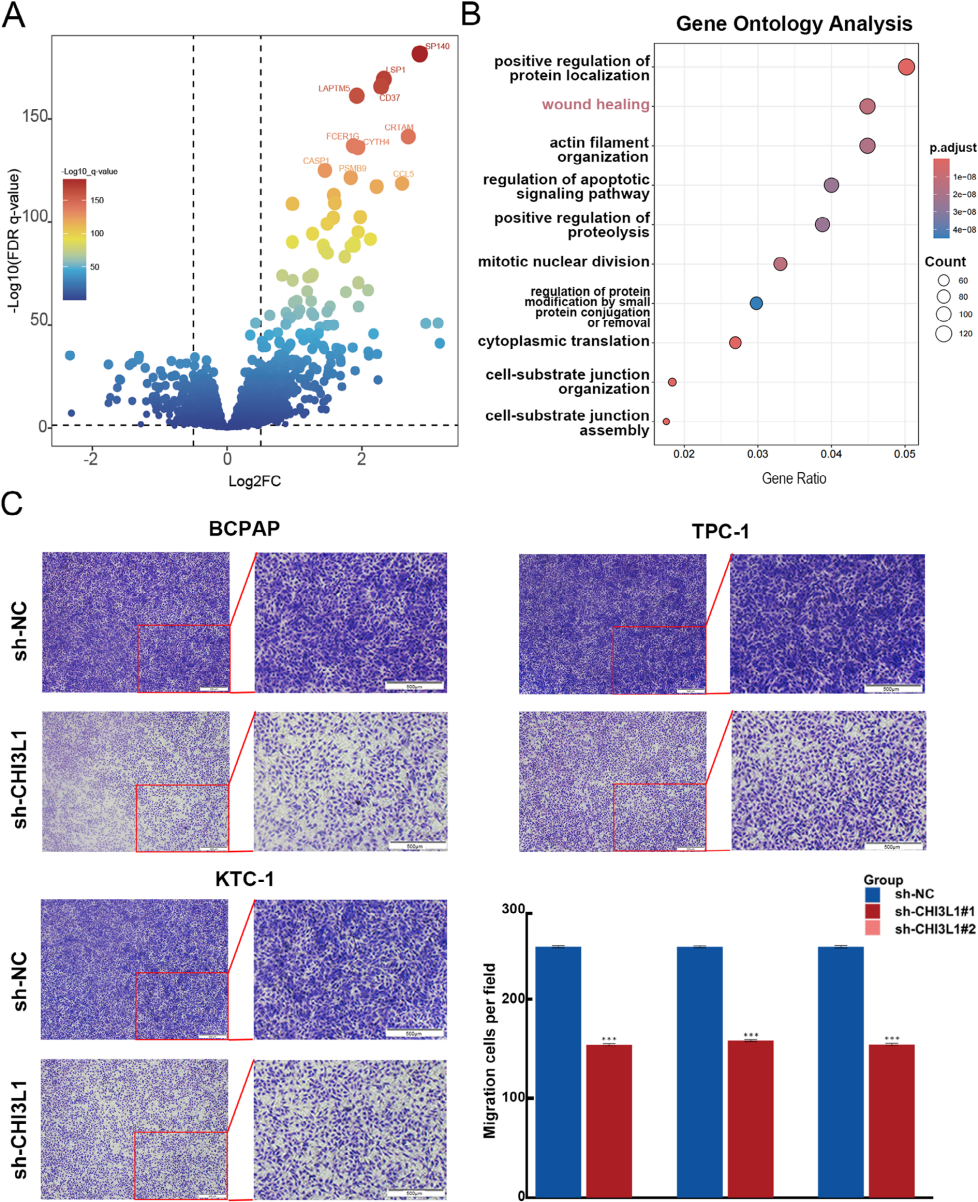
Transcriptome analysis. (A) Volcano plot highlighting differentially expressed genes. (B) Bubble chart of GO enriched pathways. (C) Transwell assay results for BCPAP, TPC‐1, and KTC‐1 lines. Scale bar in microscopy images represents 500 μm. (*p < 0.05, **p < 0.01, ***p < 0.001).

**FIGURE 5:**
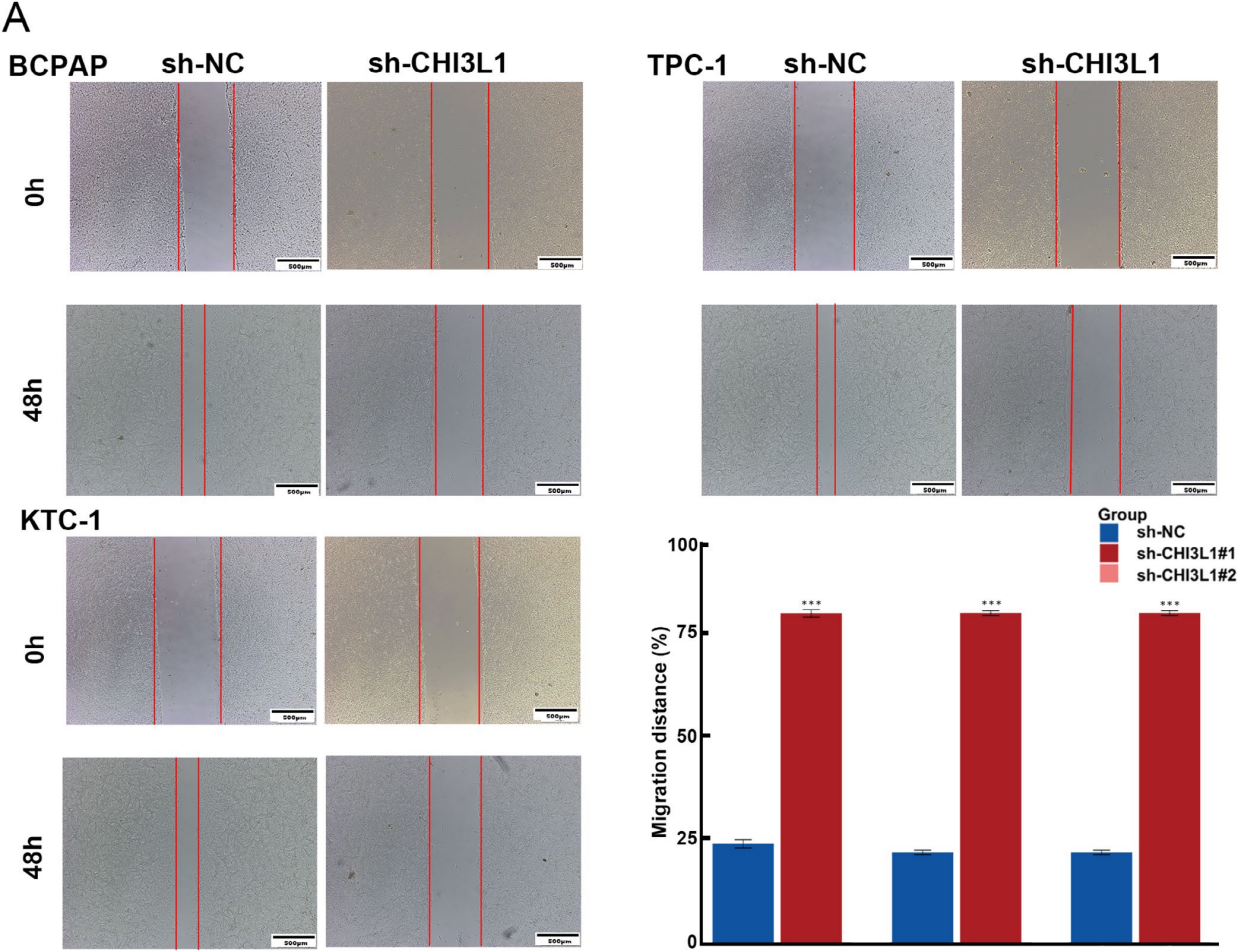
CHI3L1 regulates cell migration in thyroid cancer. (A) Wound healing assay results for BCPAP, TPC‐1, and KTC‐1 lines. Scale bar in microscopy images represents 500 μm. (*p < 0.05, **p < 0.01, ***p < 0.001).

Transcriptome sequencing using TPC‐1 cell lines identified 2911 DEGs with |log2FC| ≥ 1 and adjusted *p* value < 0.05 (Figure 4A). GO analysis of the RNA‐seq data revealed that these DEGs were associated with the wound healing pathway (Figure 4B). Functional assays corroborated these findings; specifically, transwell migration assays showed a significant reduction in cell invasion following CHI3L1 knockdown (Figure 4C). Additionally, wound healing assays confirmed a notable decrease in cell migration after CHI3L1 knockdown (Figure 5A).

### 3.4 CHI3L1 Is Associated With TP53 Signaling in Thyroid Cancer

**FIGURE 6:**
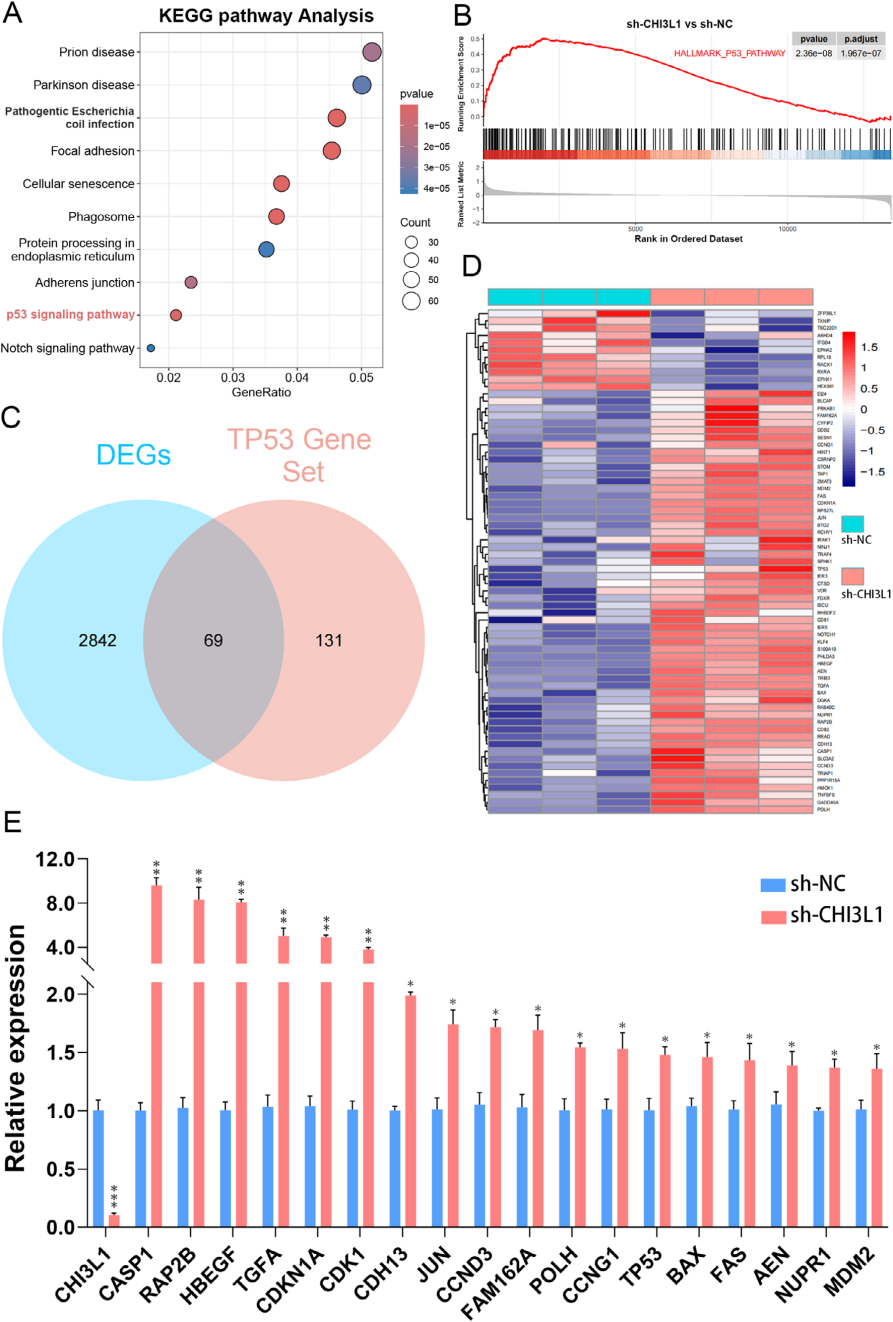
Analysis of TP53‐related gene expression following CHI3L1 knockdown. (A) KEGG enrichment analysis. (B) GSEA indicating TP53 pathway activation. (C) Venn diagram illustrating the intersection between DEGs and TP53‐related genes. (D) Heatmap of differential gene expression profiles following CHI3L1 gene knockdown. (E) Relative expression levels of selected TP53‐related genes in TPC‐1 cells after CHI3L1 knockdown. (*p < 0.05, **p < 0.01, ***p < 0.001).

To investigate the mechanism by which CHI3L1 promotes PTC, KEGG analysis of RNA‐seq data identified DEGs associated with the TP53 signaling pathway (Figure 6A). GSEA analysis further revealed significant enrichment of TP53‐related pathways in CHI3L1 knockdown groups (Figure 6B). Recent studies highlight the p53 family as a therapeutic target for aggressive thyroid cancer and autoimmune diseases [25, 26]. By intersecting the TP53 pathway gene set with the 2911 DEGs, 69 key genes involved in the PTC were identified (Figure 6C,D). Among these, 18 representative TP53‐related genes showing significant upregulation in the RNA‐seq dataset and known involvement in TP53 signaling were selected for qPCR validation in TPC‐1 cells. The results indicated significant upregulation of these genes after CHI3L1 knockdown, suggesting that CHI3L1 may influence PTC cell proliferation and migration, potentially in association with the TP53 signaling pathway (Figure 6E).

### 3.5 Association Between CHI3L1 Expression and PTC Patient Survival

**FIGURE 7:**
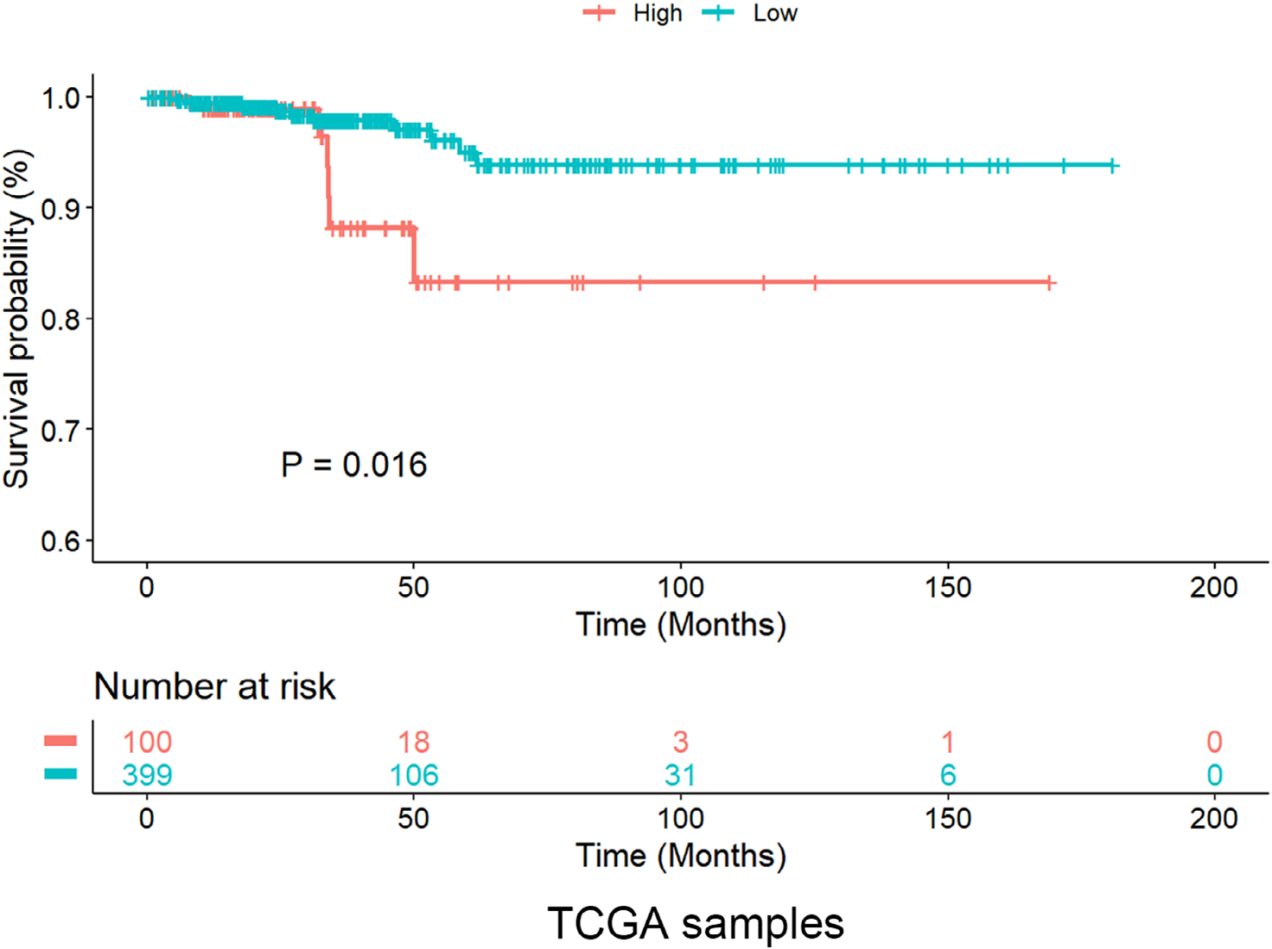
The Kaplan–Meier survival curves. Survival analysis showing reduced patient survival in patients with high CHI3L1 expression.

The TCGA database was utilized to investigate the relationship between CHI3L1 expression and the survival of patients with PTC. A total of 499 mRNA expression matrix data points for PTC, along with corresponding clinical information, were downloaded and stratified into high and low CHI3L1 expression groups. The results showed that patients with high CHI3L1 expression experienced reduced survival rates (Figure 7). These findings suggest that CHI3L1 may serve as a potential biomarker for forecasting the prognosis of PTC.

## 4 Discussion

Thyroid cancer comprises several subtypes, among which papillary thyroid cancer (PTC) is the most common and extensively studied [1, 27]. Although the prognosis for most PTC patients is favorable, advanced or radioiodine‐refractory disease remains clinically challenging. This is partly due to the complex molecular mechanisms that drive tumor progression and the shortcomings of current therapies [28, 29]. A deeper understanding of the biological drivers of PTC progression is therefore critical for the development of more effective and less invasive treatment strategies [30]. Consequently, this study aimed to elucidate the role and molecular mechanism of CHI3L1 in PTC progression.

PTC progression involves multifaceted molecular mechanisms with numerous genes, including CITED1, CHI3L1, ODZ1, N33, SFTPB, and SCEL, which are overexpressed in PTC [4]. Previous studies have demonstrated the upregulation of CHI3L1 in PTC [5], however, its precise role in PTC pathogenesis remains unclear. CHI3L1 overexpression is known to contribute to tumorigenesis in various cancers, including thyroid carcinoma, as confirmed by pan‐cancer analysis using TCGA and GTEx datasets [31, 32, 33, 34, 35]. According to a comprehensive pan‐cancer investigation of these datasets, thyroid carcinoma exhibits marked overexpression of CHI3L1, consistent with other studies that reported high expression levels in thyroid malignancies [23, 30]. A recent bioinformatics analysis reported that CHI3L1 expression was elevated in more aggressive PTC samples [24]. To further validate these findings, our differential analysis using the GEO database confirmed the elevated expression of CHI3L1 in PTC.

Furthermore, CHI3L1 overexpression was observed in both differentiated and undifferentiated thyroid cancers compared to normal thyroid tissue [36]. High levels of CHI3L1 were associated with lymph node metastases, enhanced tumor migration, and reduced patient survival in differentiated thyroid carcinoma [24, 37]. In the present study, KTC‐1, TPC‐1, and BCPAP cell lines were used for in vitro studies. In preliminary xenograft experiments, both TPC‐1 and BCPAP cells were tested for tumor formation in nude mice. However, TPC‐1 cells did not generate detectable tumors even after optimizing the injection number of TPC‐1 cells or matrigel addition. BCPAP cells were thus used for subsequent in vivo tumor formation experiments. Both in vitro and in vivo assays demonstrated that cell proliferation was suppressed when CHI3L1 was knocked down, suggesting that CHI3L1 may function as an oncogene in PTC progression.

The complex roles of CHI3L1 in tumor progression have become a growing area of research interest. Previous studies have reported that CHI3L1 was implicated in the production of inflammation and carcinogenicity, could activate the MAPK/Erk signaling pathway and contribute to tumor angiogenesis through CYR61 upregulation [14, 23, 38]. Despite these insights, little is known about the precise role of CHI3L1 in PTC. Recent studies have revealed significant differences in CHI3L1 expression among epithelial cells, suggesting its involvement in cell growth and migration pathways [39]. Our transcriptome analysis further indicated that genes differentially expressed following CHI3L1 knockdown were primarily enriched in pathways associated with migration and the TP53 signaling pathway, which were closely linked to tumorigenesis and proliferation [26, 40, 41, 42, 43]. Our experimental validation via transwell migration and wound healing assays confirmed that CHI3L1 knockdown reduces the migratory capacity of PTC cells. Moreover, this knockdown led to the upregulation of multiple TP53‐related genes, indicating a potential association between CHI3L1 and the TP53 signaling pathway in PTC.

The clinical implications of CHI3L1 in PTC prognosis are still under exploration and not yet fully understood. A recent clinical study reported that high levels of CHI3L1 in PTC biopsies may serve as a diagnostic marker [44]. Immunohistochemical analysis has also established a correlation between CHI3L1 levels and advanced clinical staging of PTC. CHI3L1 was also identified as an independent risk factor for PTC in a study using univariate and multivariate analysis, where elevated levels correlated with poorer clinical outcomes [24]. The potential of CHI3L1 as a prognostic biomarker was further supported by our survival analysis, which demonstrated that elevated CHI3L1 expression links with significantly poorer patient survival.

However, several limitations of this study should be acknowledged. First, our findings were mainly based on transcriptomic data and in vitro assays, and protein‐level validation of CHI3L1 and TP53‐related molecules was not performed. Second, validation using independent clinical tissue cohorts was lacking, which may limit the generalizability of our findings. Third, although transcriptomic analysis suggested the involvement of the TP53 signaling pathway, this observation remains largely correlative. Future studies incorporating protein‐level analysis and clinical samples will be necessary to further validate these findings.

## 5 Conclusions

In conclusion, we demonstrate that CHI3L1 is highly expressed in PTC and plays a critical role in promoting tumor progression. CHI3L1 knockdown also upregulated TP53‐related genes and suppressed tumor growth. This study highlights a novel oncogenic mechanism whereby CHI3L1 promotes tumor cell proliferation associated with downregulating the TP53 pathway. These findings provide new insights into CHI3L1's role and mechanism in PTC progression.

## Author Contributions

**Xuan Zhang:** investigation, data curation, writing – review and editing. **Yan Chang:** data curation, investigation, writing – review and editing. **Jiangqiao Geng:** conceptualization, writing – original draft, writing – review and editing, funding acquisition, supervision. **Fen Cai:** writing – original draft, data curation, visualization, validation. **Yeran Yang:** investigation, methodology, writing – review and editing, software. **Yongbo Yu:** data curation, formal analysis. **Meng Zhang:** formal analysis, data curation, writing – review and editing. **Ya'nan Zhou:** writing – original draft, data curation, methodology, software, validation. **Shengcai Wang:** methodology, writing – review and editing, resources. **Xinyuan Wu:** methodology, writing – review and editing. **Xin Ni:** conceptualization, writing – review and editing, writing – original draft, supervision. **Yongli Guo:** writing – review and editing, data curation, project administration, formal analysis.

## Funding

This work was supported by Key subjects of medical science research in Hebei Province (No. 2022180), Hebei Province 3‐3‐3 Talent Project Funding Project (No. A202105016), and the Beijing Research Ward Excel lence Program (BRWEP2024W102090103).

## Ethics Statement

The study was approved by the Beijing Children's Hospital Research Ethics Board (Ethical Approval No. 2024‐Y‐298‐D).

## Conflicts of Interest

The authors declare no conflicts of interest.

## Data Availability

The raw RNA‐seq data produced in this study have been deposited in the GEO under accession number GSE285569. Processed data are available upon request or via GSE285569 at (URL:https://www.ncbi.nlm.nih.gov/geo/query/acc.cgi?acc=GSE285569). For public data, the TCGA data was publicly available in the TCGA database (https://portal.gdc.cancer.gov/). The GTEx was publicly available at the GTEx database (https://commonfund.nih.gov/GTEx). The GSE33630, GSE27155, GSE3678, and GSE3467 datasets were publicly available in the GEO database (www.ncbi.nlm.nih.gov/geo).
